# Occurrence and distribution of tomato seed-borne mycoflora in Saudi Arabia and its correlation with the climatic variables

**DOI:** 10.1111/1751-7915.12137

**Published:** 2014-06-25

**Authors:** Abdulaziz A Al-Askar, Khalid M Ghoneem, Younes M Rashad, Waleed M Abdulkhair, Elsayed E Hafez, Yasser M Shabana, Zakaria A Baka

**Affiliations:** 1 Department of Botany and Microbiology, College of Science, King Saud UniversityRiyadh, Saudi Arabia; 2 Department of Seed Pathology Research, Plant Pathology Research Institute, Agricultural Research CenterGiza, Egypt; 3 Plant Protection and Biomolecular Diagnosis Department, City of Scientific Research and Technology Applications, Arid Lands Cultivation Research InstituteAlexandria, Egypt; 4 Science Department, Teachers College, King Saud UniversityRiyadh, Saudi Arabia; 5 Plant Pathology Department, Faculty of Agriculture, Mansoura UniversityMansoura, Egypt; 6 Botany Department, College of Science, Damietta UniversityDamietta, Egypt

## Abstract

One hundred samples of tomato seeds were collected in 2011 and 2012 from tomato-cultivated fields in Saudi Arabia and screened for their seed-borne mycoflora. A total of 30 genera and 57 species of fungi were recovered from the collected seed samples using agar plate and deep-freezing blotter methods. The two methods differed as regards the frequency of recovered seed-borne fungi. Seven fungi among those recovered from tomato seeds, which are known as plant pathogens, were tested for their pathogenicity and transmission on tomato seedlings. The recovery rate of these pathogens gradually decreased from root up to the upper stem, and did not reach to the stem apex. The distribution of tomato seed-borne fungi was also investigated throughout Saudi Arabia. In this concern, Al-Madena governorate recorded the highest incidence of fungal flora associated with tomato seeds. The impact of meteorological variables on the distribution of tomato seed-borne mycoflora was explored using the ordination technique (canonical correspondence analysis). Among all climatic factors, relative humidity was the most influential variable in this regard. Our findings may provide a valuable contribution to our understanding of future global disease change and may be used also to predict disease occurrence and fungal transfer to new uninfected areas.

## Introduction

Tomato (*Lycopersicon esculentum* Mill.) is one of the most important vegetable crops grown in Saudi Arabia. In 2011, the cultivated area under tomato in Saudi Arabia were 14 175 hectares, which produced 483 588 tons, while the annual Saudi Arabian imports of tomato were around 340 000 tons at a cost of $20 million (FAOSTAT © FAO, 2013).

Seed-borne fungi are of considerable importance due to their influence on the overall health, germination and final crop stand in the field. The infected seeds may fail to germinate, or transmit disease from seed to seedling and/or from seedling to growing plant (Islam and Borthakur, 2012). Fungal pathogens may be externally or internally seed-borne, extra- or intra-embryal, or associated with the seeds as contaminants (Singh and Mathur, 2004). Other fungi, including saprophytes and very weak pathogens, may lower seed's quality causing discolouration, which reduces the commercial value of the seeds (Elias *et al*., 2004; Al-Askar *et al*., 2012). Several fungi have been reported on tomato seeds as seed-borne in different countries (Mathur and Manandhar, 2003). *Fusarium oxysporum* is reported to be one of the most pathogenic as it can cause a 65% reduction in germination by triggering root rot and wilt of tomato. *Phoma destructiva* can reduce tomato germination by 58%, while *Alternaria solani* causes early blight of tomato (Mehrotra and Agarwal, 2003). Other seed-borne fungi that were reported on tomato include: *A. alternata, Colletotrichum gloeosporioides, Bipolaris maydis, Curvularia lunata, F. moniliforme, F. solani, F. equiseti, Cladosporium* sp.*, Aspergillus clavatus*, *A. flavus, A. niger, Penicillium digitatum*, *Pythium* sp., *Verticillium* sp., *Rhizoctonia* sp., *Rhizopus arrhizus*, *R. stolonifer* and *Sclerotinia* sp. (Nishikawa *et al*., 2006). In Saudi Arabia, reports on seed-borne mycoflora of tomato are scanty. *Alternaria alternata*, *Botrytis cinerea*, *C. herbarum*, *Drechslera* sp., *F. oxysporum*, *P. aphanidermatum*, *R. solani* and *V. albo-atrum* have been reported as seed-borne mycoflora of tomato (Al-Kassim and Monawar, 2000).

Plant pathologists have long considered environmental influences in their study of plant diseases: the classic disease triangle emphasizes the interactions between host, pathogen and environment in causing a disease (Garrett, 2008; Grulke, 2011). Climate change is just one of the many ways in which the environment can move in the long term from disease-suppressive to disease-conducive or vice versa (Perkins *et al*., 2011). Changes in environmental conditions are strongly associated with differences in the crop losses caused by a disease because the environment directly or indirectly influences growth, survival and dissemination, and hence the incidence of seed-borne fungi and the disease severity (Hudec and Muchová, 2008; Paterson *et al*., 2013). The climatic factors include rainfall, temperature*,* relative humidity and wind*.* These parameters, as they apply in air, soil or both media, also modify the transmission of seed-borne diseases. In addition, they may affect soil microflora in reduction or suppression of inoculum transfer from seed to seedling (Crowl *et al*., 2008; Eastburn *et al*., 2011), fungal growth, reproduction, survival, competitive ability, mycotoxicity and/or pathogenicity (Popovski and Celar, 2013). The present study aimed at detecting the seed-borne mycoflora of tomato, studying its distribution in the tomato-growing governorates in Saudi Arabia, and investigating the correlation between their occurrence and the climatic factors. This research is the primary investigation in a long-term project that is aiming at developing effective and eco-friendly bio-fungicides to control the most prevalent seed-borne pathogenic fungi in tomato in Saudi Arabia.

## Results

### Occurrence of tomato seed-borne mycoflora

The obtained results showed that tomato seeds were associated with a large number of seed-borne mycoflora. A total of 57 species belonging to 30 genera of fungi were recovered from the collected tomato seed samples using agar plate (AP) and deep-freezing blotter (DFB) techniques. Considerable differences were observed between the AP and DFB techniques with regard to the frequency of the recovered seed-borne fungi (Table 1). Large number of fungal species recovered from non-surface-sterilized seeds was obtained by DFB technique (26 genera and 48 species), as compared with AP method (23 genera and 44 species) (Table 1).

**Table 1:** Occurrence of tomato seed-borne fungi using agar plate (AP) and deep-freezing blotter (DFB) methods

	AP				DFB			
	Non-surface sterilized		Surface sterilized		Non-surface sterilized		Surface sterilized	
Fungus	F%^a^	I%^b^	F%	I%	F%	I%	F%	I%
*Acremonium diversisporum*	0	0	1	0.01 ± 0.01	5	0.05 ± 0.022	1	0.04 ± 0.04
*A. strictum*	1	0.01 ± 0.01	1	0.01 ± 0.01	5	0.11 ± 0.060	1	0.02 ± 0.02
*Alternaria alternata*	46	4.75 ± 1.43	21	0.54 ± 0.21	74	5.56 ± 1.36	70	4.45 ± 1.04
*A. brassicae*	0	0	1	0.01 ± 0.01	0	0	1	0.01 ± 0.01
*A. chlamydospora*	1	0.01 ± 0.01	0	0	0	0	0	0
*A. papaveris*	0	0	1	0.02 ± 0.014	0	0	0	0
*A. solani*	4	0.045 ± 0.024	4	0.055 ± 0.26	2	0.015 ± 0.011	0	0
*Aspergillus flavipes*	5	0.03 ± 0.012	4	0.025 ± 0.013	1	0.015 ± 0.015	0	0
*A. flavus*	54	2.41 ± 0.58	60	5.15 ± 0.74	14	0.12 ± 0.035	4	0.03 ± 0.016
*A. fumigatus*	2	0.015 ± 0.011	2	0.01 ± 0.007	0	0	3	0.04 ± 0.024
*A. glucus*	12	0.15 ± 0.079	10	0.1 ± 0.033	3	0.03 ± 0.019	4	0.045 ± 0.02
*A. quadrilineatus*	4	0.03 ± 0.017	0	0	10	0.055 ± 0.017	2	0.04 ± 0.02
*A. nidulans*	3	0.015 ± 0.009	3	0.03 ± 0.016	4	0.035 ± 0.022	1	0.005 ± 0.005
*A. niger*	76	6.93 ± 1.31	52	1.35 ± 0.26	26	1.13 ± 0.36	14	0.19 ± 0.052
*A. ochraceus*	10	0.1 ± 0.036	4	0.04 ± 0.02	1	0.005–0.005	1	0.01 ± 0.01
*A. tamari*	5	0.04 ± 0.022	0	0	0	0	0	0
*Aureobasidium pullulans*	65	15.74 ± 2.63	16	0.4 ± 0.14	36	1.37 ± 0.38	7	0.08 ± 0.031
*Botrytis cinerea*	0	0	0	0	2	0.04 ± 0.035	1	0.01 ± 0.01
*Cephalosporium acremonium*	5	0.06 ± 0.036	10	0.16 ± 0.06	41	1.025 ± 0.18	15	0.33 ± 0.13
*Chaetomium* spp*.*	0	0	5	0.06 ± 0.03	0	0	0	0
*Cladosporium acaciicola*	48	1.2 ± 0.3	50	1.07 ± 0.18	68	2.84 ± 0.51	72	3.51 ± 0.38
*C. cladosporioides*	28	0.85 ± 0.29	37	0.73 ± 0.17	56	1.61 ± 0.28	54	1.7 ± 0.24
*C. fulvum*	12	0.31 ± 0.2	5	0.09 ± 0.42	16	0.29 ± 0.092	23	0.43 ± 0.87
*Colletotrichum coccodes*	1	0.01 ± 0.01	0	0	3	0.02 ± 0.02	1	0.01 ± 0.01
*Curvularia lunata*	0	0	0	0	1	0.005 ± 0.005	0	0
*Drechslera australiensis*	4	0.025 ± 0.013	3	0.025 ± 0.014	2	0.01 ± 0.007	1	0.01 ± 0.01
*D. tetramera*	7	0.045 ± 0.019	0	0	2	0.02 ± 0.016	1	0.01 ± 0.01
*Emericella nidulans*	2	0.01 ± 0.007	0	0	0	0	0	0
*Epicoccum nigrum*	2	0.015 ± 0.011	3	0.045 ± 0.025	2	0.01 ± 0.007	6	0.07 ± 0.024
*Fusarium dimerum*	1	0.17 ± 0.17	1	0.01 ± 0.01	1	0.005 ± 0.005	0	0
*F. equiseti*	7	0.21 ± 0.96	0	0	15	0.6 ± 0.21	7	0.07 ± 0.028
*F. incarnatum*	3	0.15 ± 0.13	1	0.005 ± 0.005	4	0.2 ± 0.087	1	0.015 ± 0.011
*F. lateritium*	1	0.075 ± 0.075	1	0.025 ± 0.025	1	0.015 ± 0.015	0	0
*F. oxysporum*	24	2.05 ± 0.67	8	0.11 ± 0.039	18	0.41 ± 0.15	3	0.04 ± 0.23
*F. pallidoroseum*	8	0.07 ± 0.027	10	0.13 ± 0.06	6	0.055 ± 0.028	1	0.005 ± 0.005
*F. solani*	6	0.62 ± 0.39	0	0	3	0.14 ± 0.11	0	0
*F. verticillioides*	7	0.88 ± 0.82	7	0.085 ± 0.39	14	0.3 ± 0.17	0	0
*Geotrichum candidum*	38	12.53 ± 2.97	23	0.76 ± 0.23	34	0.73 ± 0.19	17	0.23 ± 0.052
*Gliocladium roseum*	0	0	0	0	1	0.01 ± 0.007	0	0
*Macrophomina phaseolina*	3	0.025 ± 0.015	0	0	7	0.12 ± 0.053	0	0
*Mucor piriformis*	25	2.78 ± 1.13	11	0.17 ± 0.06	16	1.07 ± 0.4	25	0.33 ± 0.083
*Myrothecium verrucaria*	1	0.005 ± 0.005	0	0	1	0.005 ± 0.005	1	0.02 ± 0.02
*Nigrospora oryzae*	11	0.09 ± 0.029	49	1.16 ± 0.18	1	0.005 ± 0.005	4	0.04 ± 0.02
*Penicillium polonicum*	56	3.43 ± 1.37	55	0.88 ± 0.11	45	0.95 ± 0.16	56	3.42 ± 0.62
*Phoma eupyrena*	0	0	0	0	1	0.05 ± 0.05	0	0
*P. lycopersici*	14	0.51 ± 0.23	5	0.07 ± 0.38	35	0.59 ± 0.14	17	0.26 ± 0.09
*P. medicaginis*	0	0	0	0	1	0.005 ± 0.005	0	0
*Phomopsis* sp.	0	0	0	0	1	0.005 ± 0.05	0	0
*Rhizoctonia solani*	7	0.14 ± 0.06	3	0.03 ± 0.017	3	0.03 ± 0.021	0	0
*Rhizopus stolonifer*	28	1.26 ± 0.35	31	0.67 ± 0.14	8	0.09 ± 0.034	4	0.045 ± 0.02
*Stemphylium botryosum*	11	0.09 ± 0.03	6	0.33 ± 0.58	25	0.43 ± 0.18	40	0.73 ± 0.12
*Trichoderma harzianum*	6	0.065 ± 0.03	5	0.045 ± 0.02	0	0	0	0
*Trichothecium roseum*	0	0	1	0.01 ± 0.01	0	0	1	0.06 ± 0.024
*Ulocladium alternaria*	8	0.03 ± 0.16	5	0.005 ± 0.005	24	0.035 ± 0.015	23	0.03 ± 0.021
*U. atrum*	4	0.075 ± 0.03	1	0.025 ± 0.011	6	0.22 ± 0.057	3	0.34 ± 0.9
*Verticillium dahliae*	0	0	0	0	8	0.28 ± 0.17	4	0.04 ± 0.04
*V. lecanii*	0	0	0	0	3	0.065 ± 0.045	0	0

a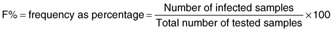.

b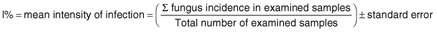.

In addition, AP technique effectively detected the seed-borne saprophytes, e.g. *A. niger* (76%), *A. flavus* (54%), *Aureobasidium pullulans* (65%), *P. polonicum* (56%) and *G. candidum* (38%). Besides, AP method succeeded to recover some fungi that were absent in DFB, e.g. *A. chlamydospora*, *A. papaveris*, *A. tamarii* and *Chaetomium* spp. (Table 1). On the contrary, the DFB technique enhanced the recovery of *C. acaciicola* (68%), *P. lycopersici* (35%), *C. cladosporioides* (56%), *C. fulvum* (16%), *A. alternata* (74%) and *Cephalosporium acremonium* (42%). Moreover, seven fungi namely, *B. cinerea*, *Gliocladium roseum*, *P. eupyrena*, *P. medicaginis*, *Phomopsis* sp., *V. dahliae* and *V. lecanii* were detected by DFB technique while AP technique was not able to detect any of them. The prevailing fungi obtained using AP method were *A. flavus*, *A. niger*, *A. pullulans*, *Geotrichum candidum*, *P. polonicum* and *R. stolonifer*, while *A. alternata*, *C. acremonium*, *Cladosporium* spp., *Stemphylium botryosum*, *Ulocladium alternaria* were the most frequent when DFB method was employed.

*Fusarium oxysporum* was the most dominant species among all *Fusarium* species (24% and 18% in both AP and DFB techniques respectively), followed by *F. equiseti* and *F. verticillioides* (15, 7% and 14, 7%, in DFB and AP techniques respectively), while *F. pallidoroseum*, *F. solani* and *F. incarnatum* were the least dominant among *Fusarium* species (8, 6%, 4, 6% and 4, 3% respectively).

In surface-sterilized seeds, high incidence of *Nigrospora oryzae* in AP, *C. fulvum* and *S. botryosum* in DFB was observed, while low incidence of *A. pullulans*, *G. candidum* and *A. niger* was recorded. On the other hand, seed surface sterilization led to complete absence of certain fungi (*F. solani* in both AP and DFB, and *F. verticillioides* and *R. solani* in DFB method).

Results of the present study showed that tomato seeds were infected with several pathogenic fungi such as *A. alternata*, *F. oxysporum*, *F. equiseti*, *F. solani*, *F. verticillioides*, *P. lycopersici, V. dahliae, Macrophomina phaseolina* and *R. solani*.

### Distribution of tomato seed-borne fungi

Seed-borne fungi varied in tomato seed samples collected from different governorates (Table 2). In this concern, Al-Madena governorate had the richest fungal diversity, recording 37 fungal species, followed by Riyadh, Tabuk, Al-Jouf and Al-kharj (33, 31, 31, 30 species respectively). On the other hand, Jeddah governorate recorded the lowest number of fungal species (19 species).

**Table 2:** Frequency percentages of tomato seed-borne fungi in tomato-growing governorates in Saudi Arabia

Fungi/Governorates	Al-Kharj	Al-Quwayiyah	Riyadh	Shagra	Wadi Al Dawasir	Al-Sulayyil	Al-Ahsaa	Al-Qatif	Najran	Gazan	Al-Ta'if	Makkah	Jeddah	Al-Jouf	Tabuk	Al-Qaseem	Hail	Al-Madenah	Fungal frequency (%)
*Acremonium diversisporum*	0.0b^a^	0.0b	0.1ab	0.0b	0.0b	0.8a	0.0b	0.0b	0.2ab	0.4ab	0.0b	0.0b	0.2ab	0.0b	0.1ab	0.0b	0.0b	0.2ab	44.44
*A. strictum*	0.0b	0.0b	0.1b	0.2b	0.0b	0.4b	0.0b	0.0b	0.0b	0.0b	0.0b	0.0b	0.2b	0.0b	0.5b	0.0b	0.0b	1.6a	38.89
*Alternaria alternata*	19.3a–c	2.3c	2.4c	3.2c	3.2c	0.3c	16.2b–c	31ab	1.8c	1.1c	0.8c	0.6c	0.4c	39.2a	0.9c	1.6c	4.6c	27.1ab	100
*A. brassicae*	0.0a	0.0a	0.0a	0.0a	0.0a	0.0a	0.2a	0.2a	0.0a	0.0a	0.0a	0.0a	0.0a	0.0a	0.0a	0.0a	0.0a	0.0a	11.11
*A. chlamydospora*	0.0b	0.0b	0.0b	0.0b	0.0b	0.0b	0.0b	0.0b	0.0b	0.0b	0.0b	0.0b	0.0b	0.0b	0.0b	0.0b	0.0b	0.1a	5.55
*A. papaveris*	0.2a	0.0a	0.0a	0.0a	0.0a	0.0a	0.2a	0.0a	0.0a	0.0a	0.0a	0.0a	0.0a	0.0a	0.0a	0.0a	0.0a	0.0a	11.11
*A. solani*	0.4a	0.0a	0.2a	0.4a	0.0a	0.0a	0.2a	0.1a	0.0a	0.1a	0.0a	0.2a	0.0a	0.0a	0.0a	0.0a	0.1a	0.1a	50
*Aspergillus flavipes*	0.0a	0.2a	0.1a	0.0a	0.2a	0.2a	0.0a	0.0a	0.3a	0.0a	0.0a	0.0a	0.1a	0.0a	0.1a	0.0a	0.0a	0.0a	38.89
*A. flavus*	7b–d	17.4a	11a–c	13.5ab	9.3a–d	6.7b–d	5.8b–d	3.7b–d	13.3a–c	6.5b–d	0.2d	0.5d	0.6d	0.1d	0.0d	0.2d	0.4d	3.5c–d	94.44
*A. fumigatus*	0.0c	0.0c	0.3ab	0.0c	0.0c	0.0c	0.0c	0.1bc	0.0c	0.0c	0.4a	0.0c	0.0c	0.0c	0.0c	0.0c	0.0c	0.1bc	22.22
*A. glucus*	0.0b	0.0b	0.4b	0.0b	0.3b	0.0b	0.3b	0.2b	0.0b	0.0b	0.6b	0.2b	0.2b	0.1b	0.4b	1.9a	0.0b	0.2b	61.11
*A. quadrilineatus*	0.0b	0.0b	0.4a	0.3ab	0.2ab	0.2ab	0.0b	0.0b	0.1ab	0.2ab	0.0b	0.2ab	0.0b	0.0b	0.0b	0.0b	0.0b	0.0b	38.89
*A. nidulans*	0.0b	0.1ab	0.5a	0.1ab	0.1ab	0.0b	0.0b	0.0b	0.2ab	0.0b	0.0b	0.0b	0.0b	0.1ab	0.0b	0.1ab	0.0b	0.3ab	44.44
*A. niger*	3bc	4.6a–c	5.1a–c	6.7a–c	1.6bc	2.2bc	4.2a–c	3.9a–c	11.7a	1.3bc	0.3c	0.5c	9.1ab	4.4a–c	11.9a	6.9a–c	6.9a–c	0.8bc	100
*A. ochraceus*	0.0b	0.0b	0.2b	0.0b	1.2a	0.2b	0.0b	0.0b	0.2b	0.0b	0.2b	0.1b	0.1b	0.1b	0.2b	0.0b	0.0b	0.2b	55.56
*A. tamarii*	0.0b	0.7a	0.0b	0.1b	0.0b	0.0b	0.0b	0.0b	0.0b	0.0b	0.0b	0.0b	0.0b	0.0b	0.0b	0.0b	0.0b	0.0b	11.11
*Aureobasidium pullulans*	62.6a	59.9ab	26c	4.3c	1.8c	1.2c	16.2c	2.9c	14.8c	24.1c	33.7bc	16.2c	5.6c	24.6c	3.1c	2.2c	2.7c	6.4c	100
*Botrytis cinerea*	0.2b	0.0b	0.0b	0.0b	0.0b	0.0b	0.0b	0.0b	0.0b	0.0b	0.0b	0.0b	0.0b	0.0b	0.0b	0.7a	0.1b	0.0b	16.67
*Cephalosporium acremonium*	0.2c	0.2c	3.4a	0.2c	0.2c	0.1c	0.4c	1.0bc	2.7ab	3.4a	0.2c	0.3c	1.0bc	0.3c	1.4bc	0.3c	0.4c	2.6ab	100
*Chaetomium* spp.	0.0b	0.0b	0.0b	0.0b	0.2ab	0.0b	0.0b	0.0b	0.2ab	0.0b	0.0b	0.0b	0.0b	0.1ab	0.6a	0.0b	0.1ab	0.0b	22.22
*Cladosporium acaciicola*	3.6bc	4.0bc	2.0c	6.6bc	2.6c	3.4bc	2.8bc	16.4a	5.7bc	1.9c	3.4bc	6.0bc	2.3c	1.4c	1.4c	2.8bc	6.5bc	9.8b	100
*C. cladosporioides*	3.4a–c	3.6a–c	1.9a–c	1.2c	1.2c	1.2c	1.4c	6.4a	4.5a–c	0.7c	1.8a–c	2.4a–c	1.8a–c	1.0c	1.7bc	3.8a–c	3.2a–c	6.1ab	100
*C. fulvum*	0.4b	0.4b	0.5b	0.4b	0.1b	0.4b	0.2b	4.6a	0.2b	0.3b	0.6b	0.6b	0.6b	0.2b	0.2b	0.2b	1.2b	2.6ab	100
*Colletotrichum coccodes*	0.0b	0.0b	0.0b	0.0b	0.0b	0.0b	0.0b	0.0b	0.0b	0.0b	0.0b	0.0b	0.0b	0.1b	0.0b	0.0b	0.0b	0.3a	11.11
*Curvularia lunata*	0.0b	0.0b	0.0b	0.0b	0.0b	0.0b	0.0b	0.0b	0.0b	0.0b	0.0b	0.0b	0.0b	0.0b	0.0b	0.0b	0.1a	0.0b	5.55
*Drechslera australiensis*	0.2a	0.0a	0.2a	0.0a	0.0a	0.0a	0.0a	0.0a	0.2a	0.0a	0.0a	0.2a	0.0a	0.2a	0.0a	0.0a	0.1a	0.1a	38.89
*D. tetramera*	0.0b	0.0b	0.4a	0.0b	0.0b	0.0b	0.1ab	0.3ab	0.0b	0.1ab	0.0b	0.0b	0.0b	0.0b	0.0b	0.0b	0.4a	0.0b	27.78
*Emericella nidulans*	0.0b	0.0b	0.0b	0.2a	0.0b	0.0b	0.0b	0.0b	0.0b	0.0b	0.0b	0.0b	0.0b	0.0b	0.0b	0.0b	0.0b	0.0b	5.55
*Epicoccum nigrum*	0.2a	0.2a	0.0a	0.2a	0.4a	0.0a	0.0a	0.0a	0.1a	0.0a	0.2a	0.1a	0.0a	0.0a	0.0a	0.2a	0.0a	0.4a	50
*Fusarium dimerum*	0.0b	0.0b	0.0b	0.0b	0.0b	0.0b	0.0b	0.0b	0.0b	0.0b	0.0b	0.0b	0.0b	0.0b	0.0b	0.0b	0.0b	3.4a	5.55
*F. equiseti*	0.1b	0.0b	0.0b	0.0b	0.0b	0.0b	0.0b	0.0b	0.0b	0.0b	0.0b	0.0b	0.0b	0.0b	1.6ab	2.5ab	0.0b	3.6a	22.22
*F. incarnatum*	0.1b	0.0b	0.2b	0.0b	0.0b	0.0b	0.0b	0.3b	0.0b	0.0b	0.0b	0.0b	0.0b	0.0b	0.0b	0.1b	0.0b	2.7a	27.78
*F. lateritium*	0.0b	0.0b	0.0b	0.0b	0.0b	0.0b	0.0b	0.0b	0.0b	0.0b	0.0b	0.0b	0.0b	0.0b	0.0b	0.0b	0.0b	1.5a	5.55
*F. oxysporum*	24.0a	5.9b	4.8b	0.6b	0.0b	0.7b	2.0b	1.0b	0.0b	0.1b	0.7b	0.1b	0.0b	0.9b	0.1b	0.2b	0.4b	1.9b	83.33
*F. pallidoroseum*	0.0b	0.0b	0.1b	0.0b	0.0b	0.0b	0.0b	0.3b	0.0b	0.0b	0.0b	0.0b	0.0b	0.3b	0.4b	0.5b	0.1b	1.5a	38.89
*F. solani*	0.1b	0.0b	0.0b	0.0b	0.2b	0.0b	0.0b	0.0b	0.0b	0.0b	0.0b	0.1b	0.0b	0.0b	0.0b	12.0a	0.0b	0.0b	22.22
*F. verticillioides*	0.6b	0.2b	0.1b	0.0b	0.1b	0.0b	0.2b	0.1b	0.2b	0.1b	0.0b	0.0b	0.0b	16.7a	1.1b	0.2b	0.0b	0.0b	61.11
*Geotrichum candidum*	0.4b	5.4b	0.3b	0.0b	0.5b	0.6b	0.6b	57.1a	0.4b	0.6b	0.4b	0.4b	0.5b	19.1b	2.2b	71.0a	5.0b	3.3b	94.4
*Gliocladium roseum*	0.0b	0.0b	0.0b	0.0b	0.1ab	0.1ab	0.0b	0.2a	0.0b	0.0b	0.0b	0.0b	0.0b	0.0b	0.0b	0.0b	0.0b	0.0b	16.67
*Macrophomina phaseolina*	0.0b	0.0b	0.6ab	0.1ab	0.0b	0.0b	0.9a	0.0b	0.5ab	0.0b	0.0b	0.0b	0.0b	0.0b	0.2ab	0.0b	0.0b	0.0b	33.33
*Mucor piriformis*	0.1c	0.2c	0.0c	0.0c	0.0c	0.0c	1.6bc	6.5b	0.2c	0.0c	0.0c	0.0c	0.0c	0.4c	0.2c	14.3a	1.0bc	4.1bc	61.11
*Myrothecium verrucaria*	0.0a	0.0a	0.0a	0.0a	0.0a	0.0a	0.0a	0.0a	0.0a	0.0a	0.0a	0.0a	0.0a	0.1a	0.0a	0.0a	0.0a	0.1a	11.11
*Nigrospora oryzae*	4.4a	1.0cd	2.2bc	1.8b–d	0.8cd	0.1d	1.0cd	0.1d	3.6ab	0.8cd	1.8b–d	0.5cd	1.2cd	0.3cd	1.9b–d	0.0d	0.1d	0.4cd	94.44
*Penicillium polonicum*	1.8cd	1.8cd	1.7cd	0.8cd	1.5cd	10.0b	3.0cd	19.4a	9.8b	5.6bc	1.7cd	0.3d	1.4cd	0.9cd	2cd	2.5cd	2.0cd	1.2cd	100
*Phoma eupyrena*	0.0b	0.0b	0.0b	0.0b	0.0b	0.0b	0.0b	0.0b	0.0b	0.0b	0.0b	0.0b	0.0b	1.0a	0.0b	0.0b	0.0b	0.0b	5.55
*P. lycopersici*	0.4b	0.2b	0.4b	0.4b	0.6b	0.2b	0.7b	0.4b	0.3b	1.6b	0.3b	0.9b	1b	5.2a	0.1b	2ab	0.2b	2.7ab	100
*P. medicaginis*	0.1a	0.0b	0.0b	0.0b	0.0b	0.0b	0.0b	0.0b	0.0b	0.0b	0.0b	0.0b	0.0b	0.0b	0.0b	0.0b	0.0b	0.0b	5.55
*Phomopsis* sp.	0.0b	0.0b	0.0b	0.0b	0.0b	0.0b	0.0b	0.0b	0.2b	0.0b	0.0b	0.0b	0.0b	1.0a	0.1b	0.0b	0.0b	0.0b	16.67
*Rhizoctonia solani*	0.0b	0.2b	0.2b	0.0b	1.2a	0.0b	0.0b	0.1b	0.0b	0.0b	0.0b	0.0b	0.0b	0.4b	0.3b	0.9ab	0.0b	0.0b	38.89
*Rhizopus stolonifer*	0.2d	1.0b–d	4.0a	3.4a–c	0.2d	0.1d	0.9b–d	1.4a–d	0.8b–d	0.3cd	0.0d	0.2d	0.4cd	0.2d	3.6ab	0.2d	0.5cd	0.7b–d	94.44
*Stemphylium botryosum*	3.5ab	2.0a–c	1.2a–c	1.2a–c	1.2a–c	0.1c	0.3c	1.9a–c	0.4c	0.2c	1.0a–c	0.2c	0.8a–c	0.2c	0.2c	0.6bc	0.2c	3.7a	100
*Trichoderma harzianum*	0.4ab	0.0b	0.6a	0.2ab	0.0b	0.0b	0.0b	0.0b	0.0b	0.0b	0.0b	0.2ab	0.0b	0.2ab	0.0b	0.0b	0.0b	0.3ab	33.33
*Trichothecium roseum*	0.4a	0.4a	0.0b	0.0b	0.0b	0.2ab	0.0b	0.0b	0.2ab	0.2ab	0.0b	0.0b	0.0b	0.0b	0.0b	0.0b	0.0b	0.0b	27.78
*Ulocladium alternaria*	2.8a	2.8a	0.4b	0.2b	0.2b	0.1b	0.2b	0.5b	0.4b	0.2b	0.2b	0.1b	0.0b	0.3b	0.2b	0.2b	0.7b	0.9b	94.44
*U. atrum*	0.3a	0.4a	0.0b	0.0b	0.0b	0.0b	0.0b	0.2a	0.1a	0.0b	0.0b	0.0b	0.0b	0.0b	0.1a	0.0b	0.0b	0.3a	38.89
*Verticillium dahliae*	0.0b	0.0b	0.0b	0.0b	0.0b	0.4b	0.0b	0.1b	0.0b	0.0b	0.2b	0.1b	0.0b	0.1b	3.1a	0.2b	0.1b	1.8ab	50
*V. lecanii*	0.0b	0.0b	0.0b	0.0b	0.0b	0.0b	0.0b	0.0b	0.0b	0.0b	0.0b	0.1b	0.0b	0.0b	0.4ab	0.0b	0.8a	0.0b	16.67

a Values followed by the same letter(s) are not significantly differed according to Duncan's multiple range test (*P* ≤ 0.05).

**Fig 1:**
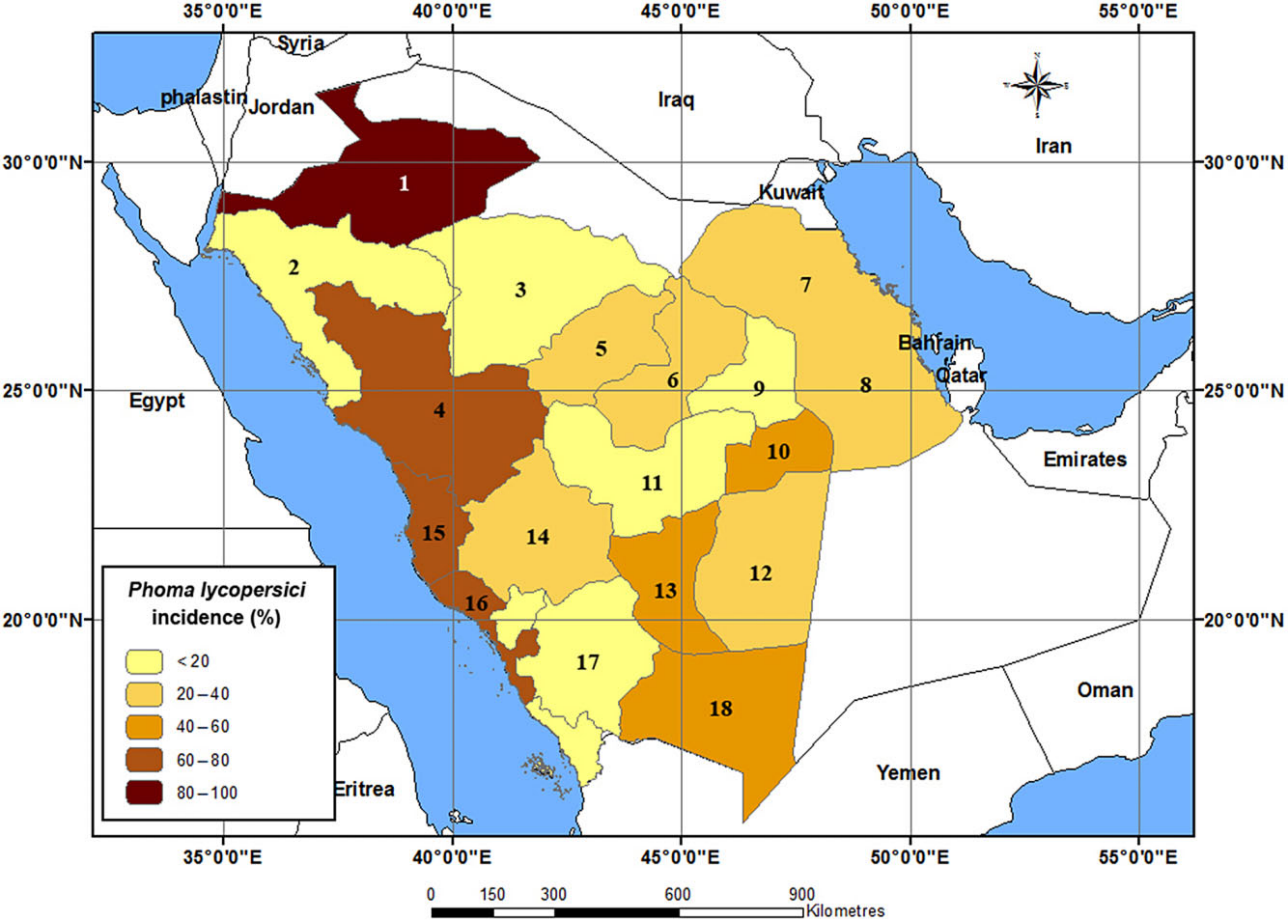
Geographical distribution of tomato seed-borne *P. lycopersici* in Saudi Arabia. The governorates names are: 1 = Al-Jouf, 2 = Tabuk, 3 = Hail, 4 = Al-Madenah, 5 = Al-Qaseem, 6 = Shagra, 7 = Al-Qatif, 8 = Al-Ahsaa, 9 = Riyadh, 10 = Al-Kharj, 11 = Al-Quwayiyah, 12 = Al-Sulayyil, 13 = Wadi Al-Dawasir, 14 = Al-Ta'if, 15 = Jeddah, 16 = Makkah, 17 = Gazan and 18 = Najran.

*Phoma lycopersici* was the most wide spread field pathogen in all tomato-growing areas of the country recording 100% frequency. Occurrence data of *P. lycopersici* were geographically mapped to show its distribution in the study area using ArcGIS 10.1 Software (Fig. 1). The highest infection intensity was recorded in Al-Jouf governorate (5.2%) and the lowest was in Tabuk governorate (0.1%).

**Fig 2:**
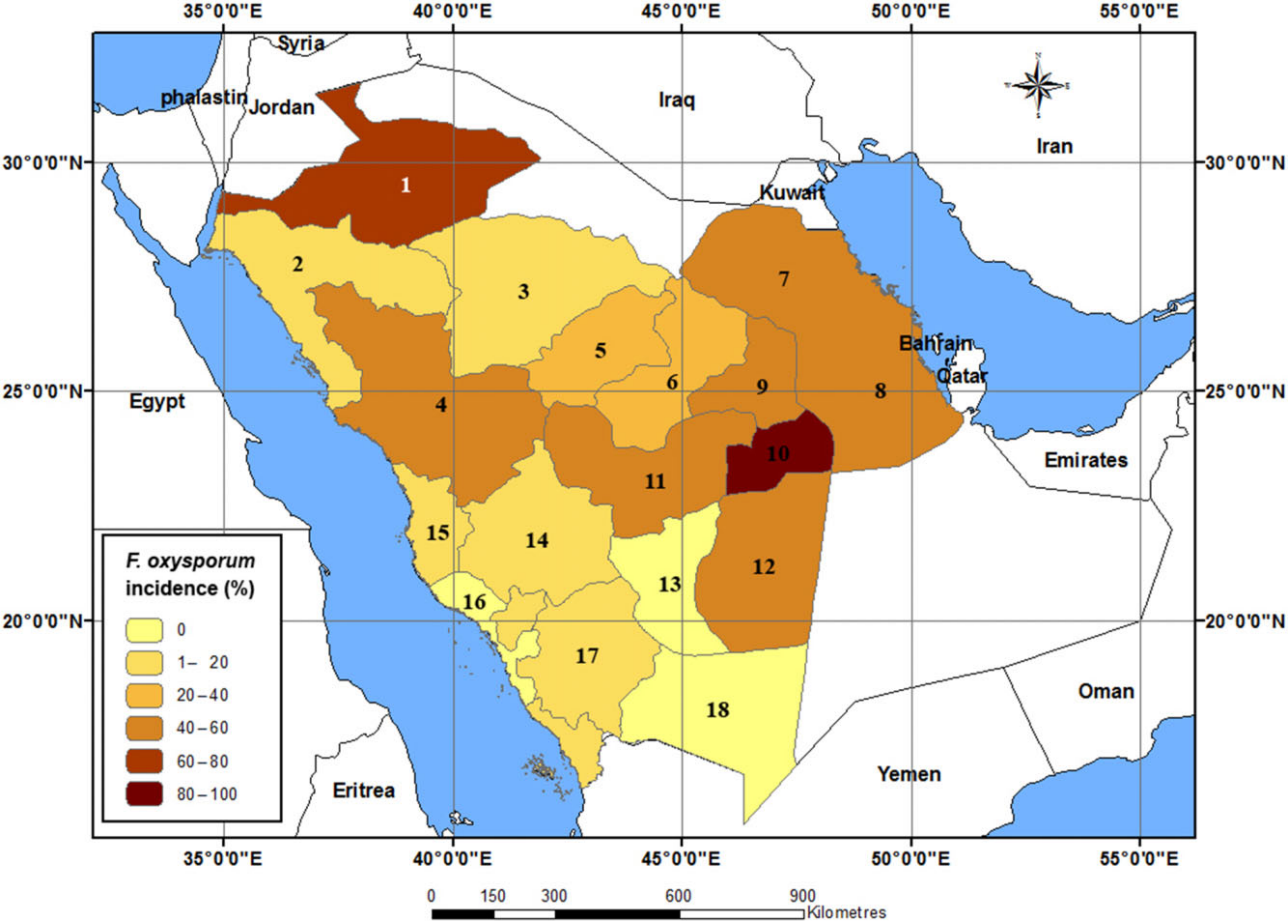
Geographical distribution of tomato seed-borne *F. oxysporum* in Saudi Arabia. The governorates names are: 1 = Al-Jouf, 2 = Tabuk, 3 = Hail, 4 = Al-Madenah, 5 = Al-Qaseem, 6 = Shagra, 7 = Al-Qatif, 8 = Al-Ahsaa, 9 = Riyadh, 10 = Al-Kharj, 11 = Al-Quwayiyah, 12 = Al-Sulayyil, 13 = Wadi Al-Dawasir, 14 = Al-Ta'if, 15 = Jeddah, 16 = Makkah, 17 = Gazan and 18 = Najran.

*Fusarium oxysporum* was the second most dominant field pathogen in the study area (83.3%). Occurrence data of *F. oxysporum* were geographically mapped to show its distribution in the study area (Fig. 2). It was found that Al-Kharj governorate recorded its highest infection intensity (24%), while the lowest infection was recorded in the seed samples obtained from Al-Qatif, Gazan, Makkah and Tabuk governorates (0.1%, for each). All sampled governorates showed moderate distribution of the field pathogens *A. solani*, *V. dahliae*, *R. solani* and *M. phaseolina* (50%, 50%, 38.9% and 33.3% respectively). Of these, *A. solani* reached its most infection intensity in Al-kharj and Shagra. *Verticillium dahliae* was the most abundant in Tabuk, while *R. solani* was the most common in Wadi Al-Dawasir and *M. phaseolina* in Al-Ahsaa. In contrast, *C. coccodes* was the least dominant field pathogen. It was found only in two governorates with very low infection intensity.

With regard to tomato post-harvest pathogenic fungi, *A. alternata*, *A. niger*, *P. polonicum* and *S. botryosum* were the most abundant in the study area, recording 100% frequency for each. The highest infection intensity for these fungi was recorded in Al-Jouf for *A. alternata* (39.2%), Tabuk for *A. niger* (11.9%), Al-Qatif for *P. polonicum* (19.4%) and Al-Madena for *S. botryosum* (3.7%). Similarly, *G. candidum*, *R. stolonifer* and *A. flavus* were found in 17 of the 18 investigated governorates. On the other hand, *B. cinerea, F. solani, F. equiseti* and *F. incarnatum* were the least abundant post-harvest pathogens recording frequencies of 16.7%, 22.2%, 22.2%, 27.8% respectively.

The obtained data showed high occurrence of saprophytic fungi in the surveyed governorates. *Aureobasidium pullulans*, *C. cladosporioides* and *C. acremonium* were the most common saprophytic fungi in the study area. They were found in all governorates. In this respect, Al-Kharj, Al-Qatif and Riyadh governorates recorded the highest incidence (62.6%, 16.4% and 3.4% respectively). On the other hand, *P. medicaginis*, *P. eupyrena*, *E. nidulans*, *F. dimerum* and *C. lunata* were the least common saprophytes associated with tomato seeds in the study area. Each fungus was found only in one governorate.

### Pathogenicity tests

Seven fungal species, i.e. *A. alternata*, *F. oxysporum, F. equiseti*, *F. solani, F. verticillioides*, *P. lycopersici* and *R. solani*, were isolated from collected tomato seed samples. They were tested for their pathogenicity on tomato seeds and seedlings. Pathogenicity tests were carried out in pots using surface-sterilized seeds of tomato. Growing-on test showed that the disease symptoms were similar in all treatments of *Fusarium* species, and were in form of rotted seeds and wilted seedlings. Infection with *A. alternata*, *R. solani* and *P. lycopersici* produced disease symptoms of leaf blight, seed rot and seedling damping-off (Table 3).

**Table 3:** Pathogenicity of fungi recovered from tomato seeds and the type of symptoms they produced under greenhouse conditions^a^

Fungus	Rotted seeds (%)	Infected seedlings (%)	Healthy seedlings (%)
Control	2.0d^b^	0	98.0a
*Alternaria alternata*	38.30b	13.0cd	48.70bc
*Fusarium equiseti*	30.0bc	10.0cd	60.0bc
*F. oxysporum*	38.60b	18.40bc	43.0c
*F. solani*	26.70bc	13.30cd	60.0bc
*F. verticillioides*	23.30c	8.40de	68.30b
*Phoma lycopersici*	28.30bc	28.40a	43.30c
*Rhizoctonia solani*	56.70a	25.0ab	18.30d

a Affected plants with different fungi in the pathogenicity test were determined during seedling stage (1–6 weeks) as: (i) Pre-emergence damping-off (rotted seeds) and (ii) Post-emergence damping-off (infected seedlings).

b Values are means of 15 replicates (pots), 10 seeds each. Values within a column followed by the same letter(s) are not significantly different according to Duncan's multiple range test (*P* ≤ 0.05).

*Rhizoctonia solani* caused the highest percentage of rotted seeds (56.7%), followed by *F. oxysporum* (38.6%), *A. alternata* (38.3%), *F. equiseti* (30%), *P. lycopersici* (28.3%), *F. solani* (26.7%) and *F. verticillioides* (23.3%) as compared with the check (2%). After 60 days, plants grown in the infested soil showed 28.4% seedling mortality due to infection of roots by *P. lycopersici*, while *R. solani* caused 25% infection. Among *Fusarium* species tested, *F. oxysporum* caused 18.4% wilting on seedlings, followed by *F. solani* and *F. equiseti* (13.3% and 10% respectively). Wilting of 13% of tomato seedlings was caused by *A. alternata*. Stems and leaves of plants become thin, dried and turned black. Two months after planting, results indicated that most tested fungi caused mild to severe infection on tomato plants. *Rhizoctonia solani* caused 81.7% mortality to tomato seedlings, while *F. oxysporum* and *P. lycopersici* exhibited seedlings mortality of 57%, followed by *A. alternata* (51.3%). Both *F*. *solani* and *F. equiseti* presented 40% infection, followed by *F. verticillioides*, which recorded seedlings infection of 31.7% as compared with check treatment.

### Transmission of seed-borne fungi in tomato plants

Tomato plants surviving the challenge of the introduced seed-borne fungi (in the pathogenicity test) were left to grow until maturity. The rate of recovery of each fungus from various plant parts, including roots, crown, basal stem (from soil surface up to 10 cm height), middle stem (from 10 to 15 cm) and upper stem (from 15 to 20 cm) and stem apex, at intervals of 60 days, was determined. Among the tested pathogens, *P. lycopersici* and *R. solani* showed the highest incidence on roots and crown parts of tomato plants (100%, 70% and 100%, 60% respectively), followed by *F. oxysporum, F. equiseti* (90%, 60% and 60%, 30% respectively). On the other hand, *F. verticillioides* and *F. solani* were restricted to root part with incidence of 80% and 50% respectively. Isolation trials from basal, middle and upper stem parts showed that *R. solani*, *P. lycopersici*, *F. oxysporum* and *F. equiseti* were restricted to basal stem part at incidence of 40%, 35%, 30% and 25% respectively. *Alternaria alternata* was the only fungus recovered from middle stem parts at infection percentage of 30%. However, the recovery percentages of the tested pathogens gradually decreased from root up to the middle stem, and none of the pathogens except *A. alternata* has reached to the middle stem (Table 4).

**Table 4:** Incidence of the pathogenic fungi in different parts of tomato plants^a^

	Incidence of fungi (%)					
Fungus	Root	Crown	Basal stem	Middle stem	Upper stem	Stem apex
Control	0.0d^b^	0.0b	0.0b	0.0b	0.0a	0.0a
*Alternaria alternata*	0.0a	60.0a	30.0a	30.0a	0.0a	0.0a
*Fusarium equiseti*	60.0bc	30.0ab	25.0a	0.0b	0.0a	0.0a
*F. oxysporum*	90.0ab	60.0a	30.0a	0.0b	0.0a	0.0a
*F. solani*	50.0c	30.0ab	0.0b	0.0b	0.0a	0.0a
*F. verticillioides*	80.0a–c	0.0b	0.0b	0.0b	0.0a	0.0a
*Phoma lycopersici*	100.0a	70.0a	35.0a	0.0b	0.0a	0.0a
*Rhizoctonia solani*	100.0a	60.0a	40.0a	0.0b	0.0a	0.0a

a Each value represents the mean of 10 replicates.

b Values within a column followed by the same letter(s) are not significantly different according to Duncan's multiple range test (*P* ≤ 0.05).

### Correlation between tomato seed-borne fungi and climatic variables

The correlation between tomato seed-borne fungi and climatic variables were analysed using canonical correspondence analysis (CCA). The eigenvalues of the two axes of the CCA are presented in Table 5. With CCA constrained to the five variables, the eigenvalues of CCA axes 1 (0.05) and 2 (0.03) explained 55.5% of the cumulative variance of the fungal species–climate relation and 14.5% of the cumulative variance of the species data (Table 5). The species–climate correlations were high (= 0.79 for both axes 1 and 2) (Table 5).

**Table 5:** Results of ordination by canonical correspondence analysis

Axis	1	2
Eigen value	0.046	0.034
Species–climate correlation	0.786	0.793
Cumulative % variance of species data	8.4	14.5
Cumulative % variance of species–climate relation	32	55.5

The correlations of climatic variables with CCA axes are defined by Table 6. Relative humidity is the only variable that is negatively correlated with axis 1 (*r* = −0.52). Axis 2 correlates positively with the temperature (*r* = 0.45), vapor (*r* = 0.45) and wind velocity (*r* = 0.55). The correlations between climatic variables are also presented in Table 6. Temperature variable is positively correlated with relative humidity (*r* = 0.45) and highly correlated with vapor (*r* = 0.71), while relative humidity is highly correlated with vapor (*r* = 0.93).

**Table 6:** Interset correlations of climatic variables with canonical correspondence analysis axes^a^

	Axis 1	Axis 2	Temperature	Relative humidity	Wind velocity	Vapor	Precipitation
Axis 1	1						
Axis 2	0.09^ns^	1					
Temperature	0.12^ns^	0.45*	1				
Relative humidity	−0.52*	0.33^ns^	0.45*	1			
Wind Velocity	0.19^ns^	0.55*	0.31^ns^	0.19^ns^	1		
Vapor	0.35^ns^	0.45*	0.71**	0.93**	−0.04^ns^	1	
Precipitation	0.35^ns^	0.32^ns^	0.14^ns^	0.05^ns^	0.19^ns^	0.02^ns^	1

**a.** ^*^Significant at *P* < 0.05; ^*^^*^^*^Significant at *P* < 0.001.

ns = not significant.

**Fig 3:**
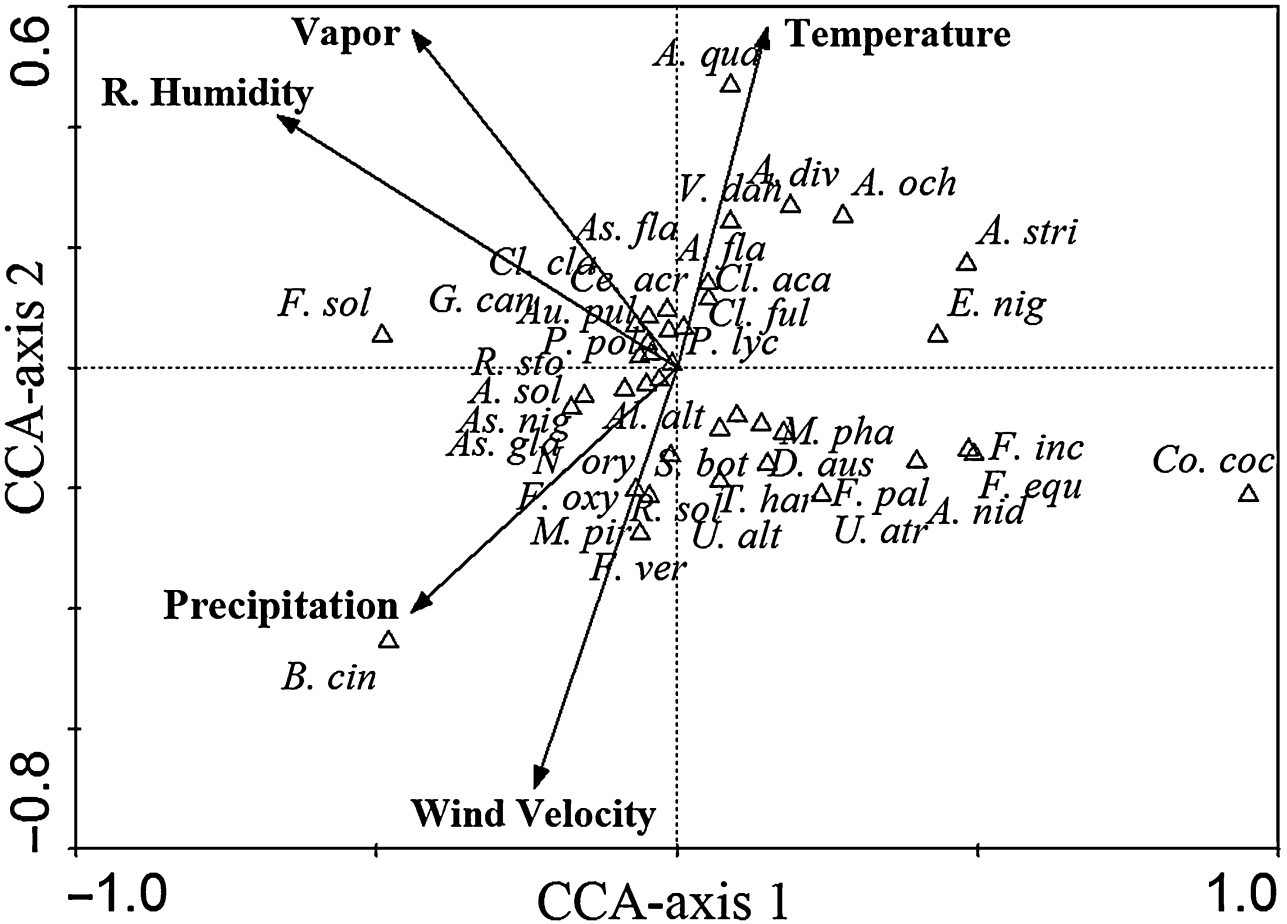
Canonical correspondence analysis (CCA) ordination diagram of the fungal species (represented by triangles) and climatic variables (represented by arrows). The fungal species names are abbreviated to first (or first two) letter(s) of the genus and first three letters of the species. The species names are: A. div = *Acremonium diversisporum*, A. stri = *Acremonium strictum*, Al. alt = *Alternaria alternata*, A. sol = *Alternaria solani*, A. fla = *Aspergillus flavipes*, As. fla = *Aspergillus flavus*, As. gla = *Aspergillus glaucus*, A. nid = *Aspergillus nidulans*, As. nig = *Aspergillus niger*, A. och = *Aspergillus ochraceus*, A. qua = *Aspergillus quadrilineatus*, Ce. acr = *Cephalosporium acremonium*, Cl. aca = *Cladosporium acaciicola*, Cl. cla = *Cladosporium cladosporioides*, Cl. ful = *Cladosporium fulvum*, Au. pul = *Aureobasidium pullulans*, B. cin = *Botrytis cinerea*, Co. coc = *Colletotrichum coccodes*, D. aus = *Drechslera australiensis*, E. nig = *Epicoccum nigrum*, F. equ = *Fusarium equiseti*, F. inc = *Fusarium incarnatum*, F. oxy = *Fusarium oxysporum*, F. pal = *Fusarium pallidoroseum*, F. sol = *Fusarium solani*, F. ver = *Fusarium verticillioides*, G. can = *Geotrichum candidum,* M. pha = *Macrophomina phaseolina*, M. pir = *Mucor piriformis*, N. ory = *Nigrospora oryzae*, P. pol = *Penicillium polonicum*, P. lyc = *Phoma lycopersici*, R. sol = *Rhizoctonia solani*, R. sto = *Rhizopus stolonifer*, S. bot = *Stemphylium botryosum,* T. har = *Trichoderma harzianum*, U. atr = *Ulocladium atrum*, U. alt = *Ulocladium alternaria* and V. dah = *Verticillium dahliae*.

The ordination diagram produced by CCA (Fig. 3) demonstrates the position of fungal species along the gradient of five climatic variables, in which points represent fungal species and arrows represent climatic variables. Climate arrows point towards the maximum change of a parameter, and arrow length indicates its importance in data interpretation. The obtained results indicated that the relative humidity is the most effective climatic variable followed by wind velocity, vapor, temperature and precipitation respectively (Fig. 3).

The fungal species *G. candidum*, *A. flavus* and *C. cladosporioides* are located in the top left quadrant of the biplot and were correlated with low levels along the relative humidity and vapor gradients. Meanwhile, the fungus *F. solani* was correlated with intermediate levels along the same gradients. In the top right quadrant of the biplot, *A. quadrilineatus* showed strong correlation along the temperature gradient. The fungal species *V. dahliae*, *Acremonium diversisporum* and *A. ochraceus* were correlated with intermediate levels along the temperature gradient, while *P. lycopersici* occupied low level along the same gradient. The fungal species *F. oxysporum*, *F. verticillioides*, *Mucor piriformis*, *A. niger*, *A. solani* and *R. solani* showed intermediate correlation with the wind velocity and precipitation gradients. Meanwhile, the fungus *B. cinerea* exhibited a close relationship with the same gradients. On the other hand, the fungal species *M. phaseolina*, *C. coccodes*, *F. incarnatum*, *F. equiseti*, *F. pallidoroseum*, *A. nidulans*, *U. atrum*, *U. alternaria*, *D. australiensis*, *T. harzianum*, *S. botryosum*, *A. alternata*, *A. solani*, *R. stolonifer*, *A. niger* and *A. glaucus* showed low correlations with the wind velocity and precipitation gradients and very low correlations with the other gradients.

## Discussion

Climate has been of great importance in the distribution of seed-borne fungi. In particular, the geographic range of a fungal pathogen is delimited by factors such as temperature, relative humidity, rainfall and wind which affect its growth, reproduction and dispersal (Boddy *et al*., 2014). In this study, we highlighted the occurrence and geographic distribution of major seed-borne fungi of tomato, and their correlations with climatic variables in Saudi Arabia.

### Occurrence and distribution of tomato seed-borne mycoflora

Seed-borne fungi are of considerable importance due to their influence on the overall health, seed germination and final crop stand in the field (Islam and Borthakur, 2012). In this connection, the obtained results showed that 57 species belonging to 30 genera of fungi were recovered from the collected tomato seed samples using AP and DFB techniques with considerable quantitative and qualitative differences between the two techniques. AP technique effectively detected the seed-borne saprophytes. This may be attributed to the stimulation effects of the nutrients in the potato dextrose agar (PDA) medium. Besides, AP method succeeded to recover some fungi that were absent in DFB. This may be due to that these fungi need external supply of nutrients that are not present in the seeds (Panchal and Dhale, 2011). Moreover, seven fungi were detected by DFB technique while AP technique was not able to detect any of them. Absence of these mycoflora in AP technique may be attributed to the antagonistic activities of the fast-growing saprophytes which were dominantly recovered in this technique. On the other hand, *F. oxysporum* was the most dominant species among all *Fusaria* species associated with tomato seeds. Similar findings were obtained by Thippeswamy and colleagues (2011) who reported that *F. oxysporum* was predominantly associated with tomato seeds*.*

Our results showed high incidence of *N. oryzae* in AP, *C. fulvum* and *S. botryosum* in DFB and low incidence of *A. pullulans*, *G. candidum* and *A. niger* after seed surface sterilization. It was suggested that *C. fulvum*, *N. oryzae* and *S. botryosum* were typically internally seed-borne as compared with the other fungi, which were presumably externally seed-borne. On the other hand, seed surface sterilization led to complete absence of certain fungi. This means that these fungi are externally seed-borne. Removal of externally seed-borne fungi by surface sterilization provided a chance for the internally seed-borne fungi to appear in greater numbers (Singh and Mathur, 2004).

Our findings revealed that tomato seeds were infected in varied degrees with several pathogenic fungi that are known to cause root rot and wilt diseases in tomato. The presence of so many pathogenic fungi at high levels in various geographical areas indicates a strong need for field surveys for these and other pathogens. There also is a serious need to increase public awareness on aspects related to seed health and to develop suitable management practices for improving the quality of the seeds. Seed health testing of major crops should be introduced in the national seed quality control system.

Results of the present study revealed that Saudi governorates varied in tomato seed mycoflora. In this concern, Al-Madena governorate had the richest fungal diversity followed by Riyadh, Tabuk, Al-Jouf and Al-kharj. The cultivated area under tomato in these governorates represents 49% of the total tomato-cultivated area in Saudi Arabia and produces about 50% of the total tomato production of the country. This may explain the high fungal biodiversity on tomato seeds samples in these governorates.

Isolation trials from basal, middle and upper stem parts showed that *R. solani*, *P. lycopersici*, *F. oxysporum* and *F. equiseti* were restricted to basal stem part, while *A. alternata* was the only fungus recovered from middle stem parts. Pathogens are either extra-embryonal or embryonal, since infection was able to cause seed rot, seedling mortality and finally death of seedlings. In this case, the pathogen may spread from seeds (primary infection) to stems, petioles and leaves. The germ tube may penetrate the host and produce local infection (e.g. *A. alternata*) or live saprophytically for a period of time, persist in a resting stage in the soil or in plant residues and infect the host at a later time (e.g. *Phoma* sp., *R. solani* and *Fusarium* spp.) (Singh and Mathur, 2004). These results are in agreement with that of Thippeswamy and colleagues (2006) who studied the location and transmission of *F. oxysporum* and *A. solani* in naturally infected tomato seeds. The results revealed that both pathogens were located in seed coat, cotyledons and in embryonic axis of tomato seedlings at various concentrations. These pathogens showed the disease cycle pattern of extra-embryal infection followed local infection.

The mechanism of seed germination may have a bearing upon the mode of transmission of inoculum from seed to seedling. In this respect, there are two types of host: epigeal in which the cotyledons are carried above ground, and hypogeal in which the cotyledons, still being covered by the seed coat. Epigeal cotyledons become green and may function like true leaves; hypogeal cotyledons remain pale and serve as storage and absorption organs. In hypogeal hosts, e.g. tomato, the fleshy cotyledons act as a starting point, often as the food base, for invasion into roots and stem of the seedling. Often these exemplify intra-embryal infection followed by either local or systemic infection (Neergaard, 1979).

### Correlation between tomato seed-borne fungi and climatic variables

With the exception of the governorates of Gazan and Najran, the study area had a desert climate characterized by extreme heat during the day, an abrupt drop in temperature at night and slight, erratic rainfall. Because of the influence of a subtropical high-pressure system and the many fluctuations in elevation, there was a considerable variation in temperature and humidity. The two main extremes in climate were felt between the coastal governorates (Jeddah and Al-Qatif) and the other interior governorates.

The correlation between tomato seed-borne fungi and climatic variables were analysed using CCA. Climate arrows in the produced ordination diagram point towards the maximum change of a parameter, and arrow length indicates its importance in data interpretation. The position of the climatic arrow depends on the eigenvalues of the axes and the interset correlations of that climatic arrow (ter Braak, 1986). The obtained results indicated that the relative humidity is the most effective climatic variable followed by wind velocity, vapor, temperature and precipitation respectively. Humidity can affect the microorganisms in different ways. Some of them tend to be more invasive to the hosts in high relative humidity, while others develop better in lower relative humidity. More frequent and abundant rainfalls, with increasing temperature and higher concentrations of water vapor, will cause favourable conditions for the development of infectious diseases (Petzoldt and Seaman, 2005; Paterson *et al*., 2013). Our findings are in agreement with that of Garrett (2008) and Eastburn and colleagues (2011) who found that fungal species respond differently to climatic variations especially humidity and temperature which are critical integral factors determining growth, survival, dissemination and hence the incidence of seed-borne fungi and disease severity. The authors reported greater influence of climatic factors, especially humidity during maturation, than the effect of genotype on seed infection level.

The obtained results indicated that the fungus *F. solani* was correlated with intermediate levels along the relative humidity and vapor gradients. However, higher incidence of fungi from the genus *Fusarium* was reported at more humid climate by other authors (Doohan *et al*., 2003). They reported that humidity/wetness and temperature are the main climatic factors affecting the development of *Fusaria* fungi, although the influence of these climatic factors is not independent of other climatic factors (Doohan *et al*., 2003). The influence of climatic conditions on the incidence of *Fusarium* species is probably both direct (e.g. an effect on mode of reproduction) and indirect (e.g. an effect of soil and vegetation types) (Popovski and Celar, 2013).

On the other hand, *A. quadrilineatus* showed strong correlation along the temperature gradient. The fungal species *V. dahliae*, *A. diversisporum* and *A. ochraceus* were correlated with intermediate levels along the temperature gradient, while *P. lycopersici* occupied low level along the same gradient. Oliveira and colleagues (2009a) studied the established correlations between fungal spore concentrations and meteorological data. They reported that *Phoma* sp. exhibited negative correlation with temperature and positive correlation with humidity. On the contrary, *Cladosporium* sp. and *Aspergillus* sp. exhibited positive correlation with temperature and negative correlation with relative humidity. On the other hand, Sanei and colleagues (2004) reported that the inoculum density of *V. dahliae* in soil showed positive correlation with temperature and relative humidity. Temperature is an important factor affecting the fungal growth and specially the bioactive enzymatic reactions in the fungal cell. Sanei and colleagues (2008) reported that temperature influenced the radial growth ratio of the isolates of *V. dahliae* and the growth response of the isolates to temperature *in vitro* was quadratic.

Due to changes in temperature and precipitation regimes, climate change may alter the growth stage, development rate and pathogenicity of infectious agents, and the physiology and resistance of the host plant (Gautam *et al*., 2013). A change in temperature could directly affect the spread of infectious disease and survival between seasons. A change in temperature may favour the development of different inactive pathogens, which could induce an epidemic. Increase in temperature with sufficient soil moisture may increase evapotranspiration resulting in humid microclimate in crop and may lead to incidence of diseases favoured under these conditions (Mina and Sinha, 2008).

Our results showed that the fungus *B. cinerea* exhibited a close relationship with wind velocity and precipitation gradients. This finding is in line with that of Oliveira and colleagues (2009b) who reported that dispersal of *B. cinerea* primarily depends on the wind to invade other uninfected fields. In the field, spores land on the host plant, germinate and produce an infection when free water from rain, dew, fog or irrigation occurs on the plant surface. Wind and rain are the primary means of dissemination of the pathogen (van der Waals *et al*., 2003). Dispersal can occur a distance from a few centimetres or less between roots in soil to hundreds of kilometres from susceptible crops. For some pathogens, long-distance dispersal is an important survival strategy enabling them to colonize new areas, survive between different seasons or affect host resistance (Wingen *et al*., 2013). The invasive potential of a pathogen can be largely explained by its ability to use atmospheric pathways for rapid spread into new areas (Viljanen-Rollinson *et al*., 2007). In a survey of fungal species associated with rainwater and atmospheric dust in Spain, Palmero and colleagues (2011) found that propagules of *F. oxysporum*, *F. verticillioides*, *F. solani*, *F. equiseti*, *F. dimerum* and *F. proliferatum* have the ability to cross continental barriers via winds and rain water deposition. The same results were achieved by Rossi and colleagues (2002) who found that peaks in spore counts of *Fusaria* fungi constantly occurred after rainfall, and the authors concluded that spore-carrying droplets originated from raindrops and remained in air currents for hours after rainfall had ceased. *Alternaria solani* and various other *Alternaria* species have been reported among few pathogens that are able to sporulate when exposed to several short wet periods interrupted by dry intervals. Fungal conidia are splashed by water or by wind onto an uninfected plant where they germinate in the presence of free water within 2 h (Aylor, 2003). This may be consistent with our finding of low correlation between *A. alternata* and *A. solani* and precipitation gradient.

In conclusion, this article provides basic information on the occurrence and geographic distribution of major seed-borne fungi of tomato, and their correlation with climatic variables in Saudi Arabia, which can be useful for setting research priorities for further disease management strategies in different agro-ecologies. It may provide a valuable contribution to our understanding of future global disease change and may be used also to predict disease occurrence and fungal transfer to new uninfected areas. Presence of different seed-borne pathogens in tomato seeds warrants for research attention in the area of seed pathology. Our results suggest that fungal biodiversity is directly affected by the climatic conditions of different locations.

## Experimental procedures

### Study area

**Fig 4:**
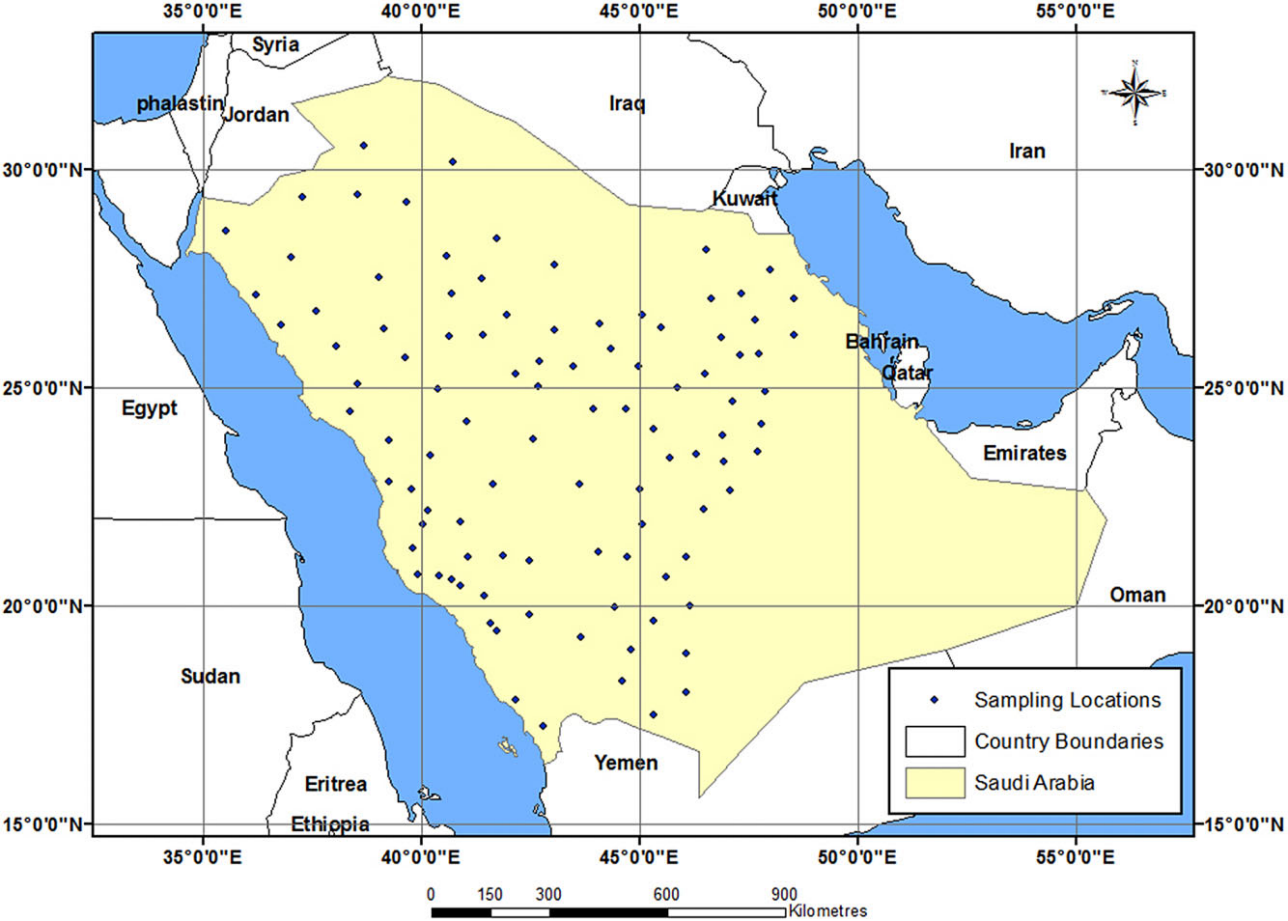
Sampling location map showing the study area in Saudi Arabia.

Tomato-growing governorates in Saudi Arabia were surveyed during 2011–2012 (all the year except July and August). The survey area lied between latitudes 17°24'N and 30°33'N, and longitudes 35°50'E and 49°11'E, as illustrated in the map in Fig. 4, which was generated using ArcGIS software, version 10.1 (Environmental Systems Research Institute (ESRI), 2012). The survey area included 18 governorates representing different climatic conditions namely, Al-Ahsaa, Al-Jouf, Al-Kharj, Al-Madenah, Al-Qaseem, Al-Qatif, Al-Quwayiyah, Al-Sulayyil, Al-Ta'if, Hail, Jeddah, Gazan, Makkah, Najran, Riyadh, Shagra, Tabuk and Wadi Al-Dawasir.

### Meteorological data

Saudi Arabia has a desert dry climate with high temperatures in most of the country. However, the country falls in the tropical and subtropical desert region. Winds reaching the country are generally dry, and almost all the area is arid. Because of the aridity and the relatively cloudless skies, there are great extremes in temperature, but there are also wide variations between the seasons and the regions (AQUASTAT, 2013). During the sampling period, the minimum air temperature varied from −2 to 28°C, the maximum was from 18 to 51°C and the average temperature was 25 ± 2°C. The relative humidity varied from 3% to 100% (mean 37.8 ± 1.0%). The rainfall average was less than 115 mm (5 inches) per year.

### Seed sampling

For each governorate, five tomato-growing fields were selected as sampling sites. The distance between two sites was at least 25 km. Locations of sampling were geo-referenced using the global positioning system. The samples were collected in a 50 × 50 m area around each sampling site in a random zigzag pattern. Full mature tomato fruits were collected in plastic bags, labeled in the field, kept on ice until reached the lab and stored at 4°C until seed extraction.

For seed extraction, tomato fruits were cut in half through the middle, and the seeds were scraped out into a plastic container using a metal spoon. The seed extract was left to sit for 2 days at room temperature while stirring the extract fluid two or three times a day until the gelatinous seed coating is starting to disappear. The seed extract was then poured through a metal kitchen strainer and washed very well with sterile water. The seeds were then spread out to dry on a porcelain plate at room temperature (25 + 2°C) for a few days. The seeds were then placed in a labeled envelope until testing.

### Detection of tomato seed-borne fungi

Detection of seed-borne fungi was done using recommended techniques by the International Seed Testing Association (Mathur and Kongsdal, 2003) namely, DFB method and AP method. A total number of 400 seeds from each sample was used. The percentage of occurrence of each fungal species recovered by each method was calculated and tabulated for comparison between the two methods.

### DFB method

The DFB method was used to detect a wide range of fungi that are able to arise easily from seeds in the presence of humidity. Non-sterilized and surface-sterilized seeds [immersed into1% Na(OCl)_2_ for 3 min] were plated in 9 cm-diameter sterile Petri dishes containing three layers of sterile blotter (filter paper) moistened with sterilized tap water at 10 seeds per Petri dish. The plates were then incubated at 20 ± 2°C for 24 h and then transferred to a –20°C freezer for 24 h. This was followed by a 5 day-period incubation at 20 ± 2°C under cool white fluorescent lights with alternating cycles of 12 h light and 12 h darkness.

### AP method

Surface-sterilized and non-sterilized seeds were plated on PDA, pH 6.5 at 10 seeds per Petri dish. The dishes were incubated at 20 ± 2°C for 7 days under cool white fluorescent light with alternating cycles of 12 h light and 12 h darkness. Seven days later, plates were examined under stereoscopic and compound microscopes to identify the retrieved fungi. Hyphal-tip and/or single-spore isolation techniques were used to obtain pure cultures of the grown fungi. All fungi were then maintained on slants of potato carrot agar for further studies.

Fungi were identified according to their cultural properties, morphological and microscopic characteristics as described by Raper and Fennel (1965), Ellis (1971), Domsch and colleagues (1980), Booth (1977), and Burrges and colleagues (1988). For determination of morphological structures, portions of fungal growth were mounted in lacto-phenol cotton blue stain on clean slides as proposed by Sime and Abbott (2002). The prepared slide was examined under a light microscope using the 40× and 100× objectives for vegetative mycelium: septation, diameters, conidiophores (sporangiophores) and the reproductive structures: conidia, sporangiospores, etc. Fungal colonies were examined under the 10× objective of the microscope. The colonial characteristics of size, texture and colour of the colony were investigated.

### Pathogenicity test

Seven fungal isolates (*A. alternata*, *F. equiseti*, *F. oxysporum*, *F. solani*, *F. verticillioides*, *P. lycopersici* and *R. solani*) were selected, as they are the most common in our survey as well as worldwide known pathogenic fungi on tomato. The fungal isolates were tested for their pathogenicity using soil infestation technique. Each fungal isolate was cultured on maize meal substrate containing 5% soil and 15% moisture for 2 weeks at 26 ± 2°C. Plastic pots (20 cm-diameter) filled with steam-sterilized soil were infested singly with the fungal inocula at the rate of 0.4% w/w, mixed thoroughly, then regularly watered to near field capacity with tap water. Control pots filled with steam-sterilized soil received water only. Physical and chemical characteristics of the used soil are presented in Table 7. Healthy seeds of tomato (cv. Red Gold) were disinfected by immersing them in 1% sodium hypochlorite solution for 3 min, thoroughly washed three times with sterilized water, and plot dried on sterilized tissue paper. Ten surface-sterilized seeds were sown in each pot (1 week after soil infestation with the fungi) and replicated 10 times. All pots were arranged in a complete randomized design and kept under greenhouse conditions (day temperature 25 ± 3°C, night temperature 20 ± 3°C and 16 h photoperiod) for 60 days. Daily observations for germination and symptoms of pre- and post-emergence damping off were recorded. Data on pre-emergence damping off (% rotted seeds), post-emergence damping off (% infected seedlings) and plant survival were recorded.

**Table 7:** Physical and chemical characteristics of the soil used in the pathogenicity test

Physical characteristics		Chemical characteristics	
Texture	Loam	CaCO_3_ (%)	4.52
Sand (%)	42	Organic matter (%)	0.94
Clay (%)	26	N (mg. kg^−1^)	46.9
Silt (%)	32	P (mg. kg^−1^)	4.15
Electrical conductivity (dS.m^−1^)	1.13	K (mg. kg^−1^)	278.5
pH (1:2.5 soil : water)	7.92	Exchangeable sodium percentage (%)	52.5

### Transmission of seed-borne fungi in tomato plants

Tomato plants surviving the challenge of the seed-borne fungi in the previous test were allowed to grow until maturity. Every 2 months, 20 plants were pulled from pots, washed, disinfected and dissected under sterile conditions. The various plant parts (roots, hypocotyls, basal stem, middle stem, upper stem, flowering branch top, inflorescence, flowers and seeds, if present) were plated on PDA and incubated at 24 ± 2°C under cool white fluorescent light with alternating cycles of 12 h light and 12 h darkness for 7 days. Fungi recovered from each part were identified and the transmission rate and percentage were calculated.

### Statistical analysis

Comparison of means was performed with Duncan's multiple range test (Duncan, 1955) at *P* ≤ 0.05 using the statistical analysis software ‘CoStat 6.4’ (CoStat, 2005). The correlation between tomato seed-borne fungi and climatic variables are indicated on an ordination diagram produced by CCA using CANOCO program (ver. 4.51) (ter Braak, 1988).
